# Does dietary folic acid supplementation in mouse NTD models affect neural tube development or gamete preference at fertilization?

**DOI:** 10.1186/s12863-014-0091-x

**Published:** 2014-08-27

**Authors:** Ghunwa A Nakouzi, Joseph H Nadeau

**Affiliations:** 1 Department of Genetics, Case Western Reserve University School of Medicine, Cleveland, OH, USA; 2 Present address: Center for Human Genetics, University Hospitals, Cleveland, OH, USA; 3 Present address: Pacific Northwest Research Institute, 720 Broadway, Seattle 98122, WA, USA

**Keywords:** Neural tube defects, Mouse models, Folic acid, Apob, Vangl2, Embryonic lethality, Polyamines, Fertilization, Gametes, Sperm, Oocyte, Epigenetics

## Abstract

**Background:**

Neural tube defects (NTDs) are the second most common birth defect in humans. Dietary folic acid (FA) supplementation effectively and safely reduces the incidence of these often debilitating congenital anomalies. FA plays an established role in folate and homocysteine metabolism, but the means by which it suppresses occurrence of NTDs is not understood. In addition, many cases remain resistant to the beneficial effects of folic acid supplementation. To better understand the molecular, biochemical and developmental mechanisms by which FA exerts its effect on NTDs, characterized mouse models are needed that have a defined genetic basis and known response to dietary supplementation.

**Results:**

We examined the effect of FA supplementation, at 5-fold the level in the control diet, on the NTD and vertebral phenotypes in *Apob*^*tm1Unc*^ and *Vangl2*^*Lp*^ mice, hereafter referred to as *Apob* and *Lp* respectively. The FA supplemented diet did not reduce the incidence or severity of NTDs in *Apob* or *Lp* mutant homozygotes or the loop-tail phenotype in *Lp* mutant heterozygotes, suggesting that mice with these mutant alleles are resistant to FA supplementation. Folic acid supplementation also did not affect the rate of resorptions or the size of litters, but instead skewed the embryonic genotype distribution in favor of wild-type alleles.

**Conclusion:**

Similar genotypic biases have been reported for several NTD models, but were interpreted as diet-induced increases in the incidence and severity of NTDs that led to increased embryonic lethality. Absence of differences in resorption rates and litter sizes argue against induced embryonic lethality. We suggest an alternative interpretation, namely that FA supplementation led to strongly skewed allelic inheritance, perhaps from disturbances in polyamine metabolism that biases fertilization in favor of wild-type gametes.

## Background

Neural tube closure is an early developmental process that gives rise to the central nervous system, including the spinal cord and brain [1],[2]. Failure of the neural tube to close properly leads to different clinical types of NTDs depending on the site and timing of closure failure [1]–[6]. Neural tube defects (NTDs) are serious and common birth defects resulting from both genetic and environmental factors [1]–[6]. In humans, FA supplementation of maternal diet before and during pregnancy significantly reduces NTD incidence [1]–[8].

FA plays a role in both the folate cycle for the production of thymidylate and purines mediating cell division, and in the methylation cycle of homocysteine metabolism resulting in epigenetic regulation of gene expression [9]–[11]. Although the efficacy of FA supplementation is widely accepted, the mechanism by which FA reduces the incidence of NTDs is not understood and whether FA-resistant cases respond to alternative dietary nutrients is not generally known. Several studies have implicated FA in reproduction and fertility in humans [12]–[17] as well as with developmental delay and increased rates of cardiac defects and other fetal anomalies in mouse models [18]–[20], suggesting that the effects of folate metabolism and FA supplementation on pregnancy and gamete biology may be more diverse than generally appreciated.

Mouse NTD models with specific responses to different nutritional supplements can be used to study mechanisms of FA responsiveness in humans and mice, and to identify alternative approaches to prevent FA-resistant NTDs [21]–[23]. In particular, mouse models involving known genes, characterized mechanisms, and established responses to FA supplementation are needed. However, among more than 240 NTD mouse mutants and strains, only 19 have been tested for response to FA supplementation on the outcome of NTDs, with supplementation effective in some mutants but not others [2],[23]–[26]. Our lab sought to expand this body of knowledge by studying the effect of FA on selected NTD mouse models.

**Figure 1:**
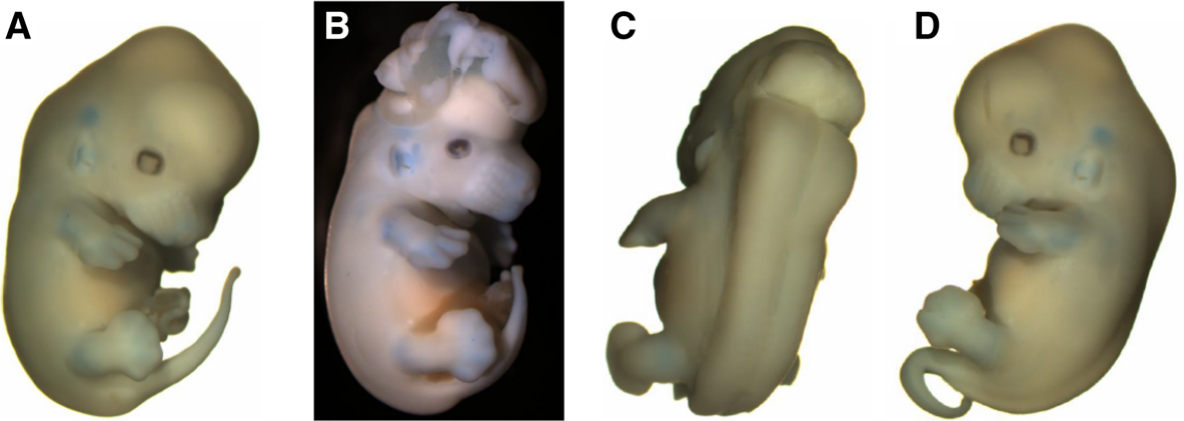
**Examples of congenital defects in** ***Apob*** **and** ***Lp*** **mutant mice. A**. Normal embryo, **B**. *Apob* – exencephaly, **C**. *Lp* – craniorachischosis, and **D**. *Lp* – loop-tail.

We examined the FA response of two NTD mouse mutants. Apolipoprotein B (apoB) is a key structural component of several lipoproteins that transport circulating cholesterol, lipids, and vitamin E [27]. The *Apob*^*tm1Unc*^ mutant is the result of a genetically engineered loss-of-function (LOF) mutation in the *Apob* gene [27]. *Apob* homozygous embryos show a 30% penetrance of exencephaly alone or accompanied with hydrocephalus [27], see also [28] (Figure 1A vs B). By 8 weeks of age, mutants that have a closed neural tube show hydrocephalus in 32% of homozygotes and in 1% of heterozygotes. VANGL2 protein is one of two highly conserved membrane proteins involved in establishing planar cell polarity (PCP) and in regulating convergent extension movements during embryogenesis [29]. The *Vangl2*^*Lp*^ mutant results from a spontaneous LOF mutation in the *Vangl2* gene [29], see also [28]. *Lp* homozygous embryos have a 100% penetrance of craniorachischisis due to failure to initiate neural tube closure at embryonic day E8.5 [30] (Figure 1A vs C). This mutation is inherited in a co-dominant manner and the heterozygous phenotype is characterized by a looped tail resulting from vertebral anomalies [29] (Figure 1A vs D). Neither mutant has been previously tested for response to dietary FA supplementation.

During our work on the effects of dietary FA supplementation on mouse models of NTDs, we made an observation that others had made with other NTD models, but were led to an alternative interpretation that seems more consistent with the entire body of data. In particular, we found that parental FA supplementation did not reduce the incidence or severity of NTDs in these two mouse models, but instead caused a substantial deficiency in the numbers of homozygous and heterozygous mutant embryos, without a corresponding increase in resorptions or a reduction in litter size. We suggest that FA supplementation led to preferential fertilization and biased segregation in heterozygous mutant mice. Obviously more work is needed to characterize molecular mechanisms, but we thought an initial report was appropriate to highlight this issue.

## Results

We began by testing whether parental FA supplementation reduced the incidence or severity of NTDs in homozygous mutant embryos or the loop-tail phenotype in *Lp* heterozygous mutant mice. Timed-pregnancies were generated with females that were either supplemented with FA (10 ppm) or maintained on a baseline FA diet (2 ppm) before mating and during pregnancy. Homozygous *Apob* and *Lp* embryos were examined for NTDs [27]–[29], see also [31]–[33] and *Lp* mutant heterozygotes for the loop-tail phenotype (Figure 1). In particular, the proportion of affected embryos did not differ between the two test and control groups (Table 1), suggesting that these NTD mutants are resistant to the beneficial effects of dietary FA supplementation.

**Table 1:** Association between parental FA supplementation and incidence of NTDs

**Mutant, diet**	**% Affected (n)**	**Sample size**
*Apob*^*tm1Unc/tm1Unc*^ - exencephaly		
2 ppm	96 (26)	27
10 ppm	96 (26)	27
*Vangl2*^*Lp/Lp*^ - craniorachischisis		
2 ppm	100 (15)	15
10 ppm	100 (11)	11
*Vangl2*^*Lp/Lp*^ – looped tail		
2 ppm	98 (40)	41
10 ppm	97 (31)	32

Fisher’s exact test was used to determine whether FA-supplementation affected the incidence of NTDs in mutant homozygotes or heterozygotes. The number of affected embryos is shown in parentheses. No significant differences were detected.

**Figure 2:**
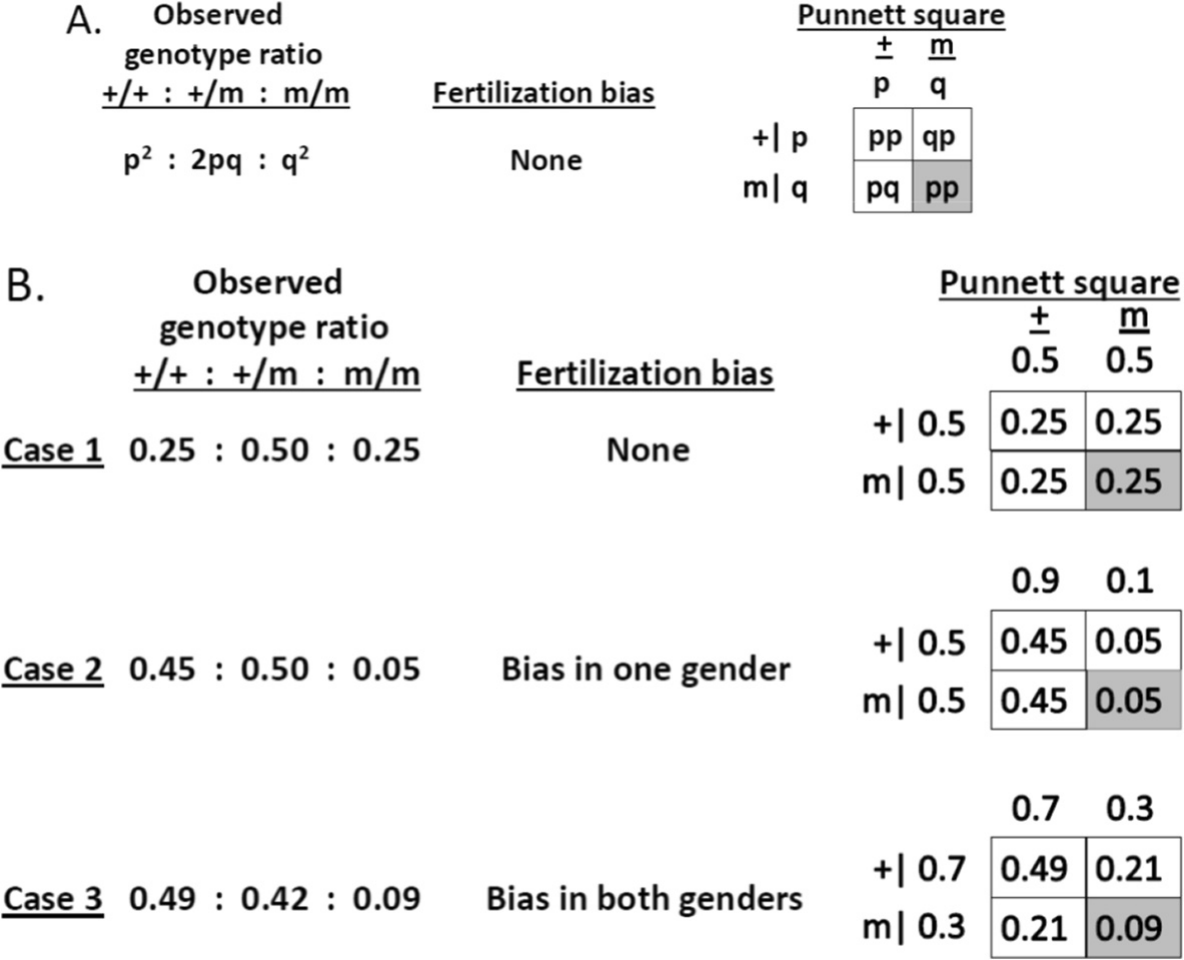
**Gamete bias at fertilization and conceptus genotype frequencies.** ‘+’ and ‘m’ designate gametes that carry the wild-type or the mutant allele, respectively. Gamete frequencies are shown on the sides of the matrix, and conceptus genotype in the cells of the matrix. Each side of the matrix represents one of sexes in each mating. **A**. General case, where p and q denote alternative alleles. **B**. Arbitrary numbers were used to illustrate the consequences of gametic bias. Note that all eggs are fertilized and litter size remains unchanged in each scenario; only the genotypic ratio changes.

Unexpectedly, both supplemented lines showed a substantial deficiency of homozygous and heterozygous mutant embryos at the higher FA concentration. Because these single gene mutations are inherited in a Mendelian manner [27]–[29], 25% of the embryos are expected to have a wild-type genotype (+/+), 50% a heterozygous genotype (+/mutant), and 25% a homozygous mutant genotype (mutant/mutant) (Figure 2). The genotype distribution on the control diet was consistent with Mendelian expectations for both mutants, showing that segregation was normal at 2 ppm FA. By contrast, a clear deficiency was found for homozygous and heterozygous embryos conceived and maintained on 10 ppm FA (Table 2). For the supplemented *Lp* mutant, although the deviation from Mendelian ratios was not statistically significant, the observed numbers of homozygous and heterozygous mutant embryos was strongly reduced relative to expectations, with the percent difference comparable to results for the *Apob* mutant, but with a slightly smaller sample size (Table 2).

**Table 2:** Embryo loss among progeny of NTD heterozygous mutant intercrosses

**Mutant, Diet**	**Obs. no. embryos**			**P-value (χ**^**2**^**)**	**% Lost heterozygous, homozygous**	**% Lost (combined)**	**% resorbed (n)**	**Ave. litter size (n)**
	**+/+**	**+/−**	**−/−**					
***Apob***^***tm1Unc***^								
2 ppm	24	48	27	ns	--	--	12.3 (14)	6.2 (16)
10 ppm	40	**38**	**27**	0.004 (11.2)	52.5, 32.5	45.8	7.8 (9)	5.8 (18)
***Vangl2***^***Lp***^								
2 ppm	13	41	15	ns	--	--	9.2 (7)	4.3 (16)
10 ppm	23	**32**	**11**	0.11 (4.4)	(30.4, 52.2)	37.7	9.6 (7)	4.4 (15)

All embryos were genotyped. Only genotyped embryos were included in litter size metrics. Resorptions were not genotyped. Chi-square goodness-of-fit tests were used to determine whether the observed genotypic distribution of embryos deviated significantly from Mendelian expectations (1:2:1) for the two NTD models and for the two FA diets. The P-value for this test is provided. To calculate the percentage of “embryo loss”, we assumed that the observed number of wild-type embryos was the correct number for the 1:2:1 Mendelian distribution. From this, we estimated expected numbers of heterozygous and mutant homozygous embryos, and then calculated the difference between the expected and observed numbers. The percent embryo loss was calculated for *Apob* (10 ppm) where genotyping results differed significantly from Mendelian expectations (bold numbers). The percent embryo loss is also provided in parentheses for *Vangl2* where a strong but non-significant trend was found. Note that differences in resorptions and litter size did not account for percent embryo loss. Bold numbers highlight results of particular interest. ns, not significant; na, not applicable.

Finally, we sought to estimate the number of missing embryos. Because FA supplementation is not expected to affect the number of wild-type embryos, we accepted the number of +/+embryos as indicative of Mendelian expectations. By extrapolation, we then estimated the expected number of heterozygous and homozygous mutants cf. [24],[25]. This analysis assumed that fertilization was random with respect to the genetic constitution of gametes in both parents. We found that on the 10 ppm diet, 52.5% and 32.5% of the expected numbers of *Apob* mutant heterozygotes and homozygotes were missing, respectively (Table 2). Similarly, 30.4% and 52.2% of the expected numbers *Lp* mutant heterozygote and homozygote embryos were missing on the 10 ppm diet. Interestingly, we found no evidence for increased rates of congenital anomalies among heterozygous mutant embryos on the supplemented diet, implying that a substantial number of phenotypically normal heterozygous mutant embryos were missing (Table 2). Finally, we noted that average litter size did not differ between test and control crosses. We also counted the number of resorptions as a measure of fetal loss, but these counts did not differ. Thus a substantial number of embryos appeared to be missing, with no evidence for embryonic lethality.

## Discussion

Understanding the molecular and developmental mechanisms by which dietary supplementation affects neural tube development is critical to reducing the impact of one of the most common birth defects, especially since some NTDs appear to be resistant to the beneficial effects of FA [1]–[6],[21]–[23]. Animal models are essential for studying experimentally the ways that these dietary factors modulate protein functions, biochemical pathways, and developmental processes during neural tube formation [21]–[26],[28],[34]. In the present study, we found that parental FA supplementation did not protect embryos either from exencephaly in *Apob*^*−/−*^ embryos or from craniorachischisis and looped-tail phenotypes in *Lp*^*−/−*^ and *Lp*^*+/−*^ embryos (Table 1). Embryos exposed to the test diet that had 5-fold more FA than the control diet did not show significant changes in the incidence or severity of defects. Thus FA supplementation did not beneficially impact aspects of lipid transport (*Apob*) and planar cell polarity (*Vangl2*) in these two mutant mice.

Unexpectedly, we found strongly biased genotype distributions with folate supplementation in both mutants, but without reduced litter size, increased resorption rates, or other evidence for differences in embryonic viability. We reviewed the literature to determine whether similar genotypic deviations without embryo loss had been reported in other NTD diet-supplementation studies. Responses of several NTD models to various nutrients have been tested, with some showing responsiveness and others resistance to supplementation or to deprivation [21]–[26],[28],[34]. Data in some reports are consistent with normal Mendelian segregation in both test and control groups [18]–[20],[23],[25],[34]. Remarkably, at least two studies involving three NTDs models also report non-Mendelian segregation (Table 3). The proportion of missing embryos was similar among models and studies, with the observed genotypic deviations corresponding to a ~20% - ~70% reduction in the number of both heterozygous and homozygous mutant embryos (Table 3). In particular, embryo loss as a function of Mendelian expectations for homozygous mutants ranged from 43% for *Zic2* to 70% for *Lrp6*, and for heterozygous mutants from 22% for *Lrp6* to 29% for *L3P*. Interestingly, corresponding changes in resorptions rates and litter sizes were not found.

**Table 3:** Embryo loss among progeny of NTD mutant intercrosses

**Mutant, Diet**	**Obs. no. embryos**			**P-value (χ**^**2**^**)**	**% Lost heterozygous, homozygous**	**% Lost (combined)**	**% Resorbed (n)**	Ave. litter size** (n)	Reference*
	**+/+**	**+/−**	**−/−**						
***Lrp6***^***ko***^									
2 ppm	37	73	20	0.04 (6.4)	1.4, 45.9	na	18 (29)	7.9 (20; 4, 12)	1
10 ppm	55	**86**	**16**	0.0001 (20.8)	21.8, 70.1	38.2	16 (30)	8.5 (22; 2–12)	1
***Zic2***									
2 ppm	56	**84**	**32**	0.03 (6.8)	25.0, 42.9	31.0	7.5 (14)	6.1 (28)	2
10 ppm	38	98	36	ns	na	na	11.8 (23)	5.7 (30)	2
***L3P***									
2 ppm	28	**40**	**11**	0.03 (7.3)	28.6, 60.7	39.3	15.9 (15)	7.2 (13)	2
10 ppm	14	28	16	ns	na	na	18.3 (13)	4.2 (10)	2

See Table 2 for details. ns – not significant, na – not applicable. (Revised from Gray et al. [24] and Marean et al. [25], with permission of the publishers) Bold numbers highlight results of particular interest.

*References: (1) Gray et al. [24], (2) Marean et al. [25]. ** Based on genotyped embryos only.

Significant departures from Mendelian expectations without embryo loss may be a regular but overlooked finding in NTD diet supplementation studies. Previously 19 models were tested for response to supplementation. The present study brings the total to 21. Of these five (*Lrp6*, *Zic3*, *L3P*, *Apob* and *Vangl2*) show non-Mendelian segregation (Tables 2 and 3), suggesting that skewed genotype ratios without embryonic lethality may be common. Treatment protocols differ among studies with supplementation in some cases introduced before conception e.g. [18]–[20],[23]–[25] and in other cases during gestation e.g. [23],[33],[34]. Only the former protocol tests for effects of supplementation on Mendelian segregation.

Deficiency of particular genotypic classes is usually interpreted as diet-induced lethality among genetically predisposed embryos [24],[25]. Various evidence argues against this interpretation. For example, fertilization of “reserve” wild-type oocytes might compensate for missing conceptuses. But with few exceptions, all ovulated oocytes are fertilized and litter size is closely related to the number of ovulated eggs. Results for heterozygotes are also particularly interesting because these mice usually show full viability, with *Vangl2*^*Lp/+*^ only showing a looped tail and *Apob*^*tm1Unc/+*^ heterozygotes appearing phenotypically normal [27],[29]. We found no evidence for FA-induced congenital anomalies among surviving heterozygous embryos. Diet-induced anomalies are occasionally reported, e.g. an NTD in a single *Pax3*^*Sp2H/+*^ heterozygote that had been exposed to thymidine supplementation during gestation [23]. But these cases are exceptional and loss of substantial numbers of phenotypically normal mutant heterozygotes with dietary supplementation is therefore perplexing.

The epidemiological evidence for folate effects is largely based on differences in NTD occurrence in supplemented versus unsupplemented pregnancies [1]–[8]. Genetic tests are rarely included in these population studies because the genetic basis is not known for most NTD cases [1],[6]. Hence the inference is made that a change in NTD occurrence results from beneficial effects of folate action on development of the neural tube, rather than a change in the occurrence of NTD-susceptible genotypes in FA supplemented populations.

We propose an alternative interpretation, namely that FA supplementation biases the combination of gametes that join to form a conceptus. Preferential fertilization would change the genotype distribution among conceptuses without reducing litter size or inducing embryonic lethality (Figure 2). We note that biased segregation was found only in intercrosses, and not in backcrosses to wild-type (GAN and JHN, unpubl.), suggesting a preference for specific combinations of sperm and oocyte at fertilization, rather than intrinsic gametic defects.

**Figure 3:**
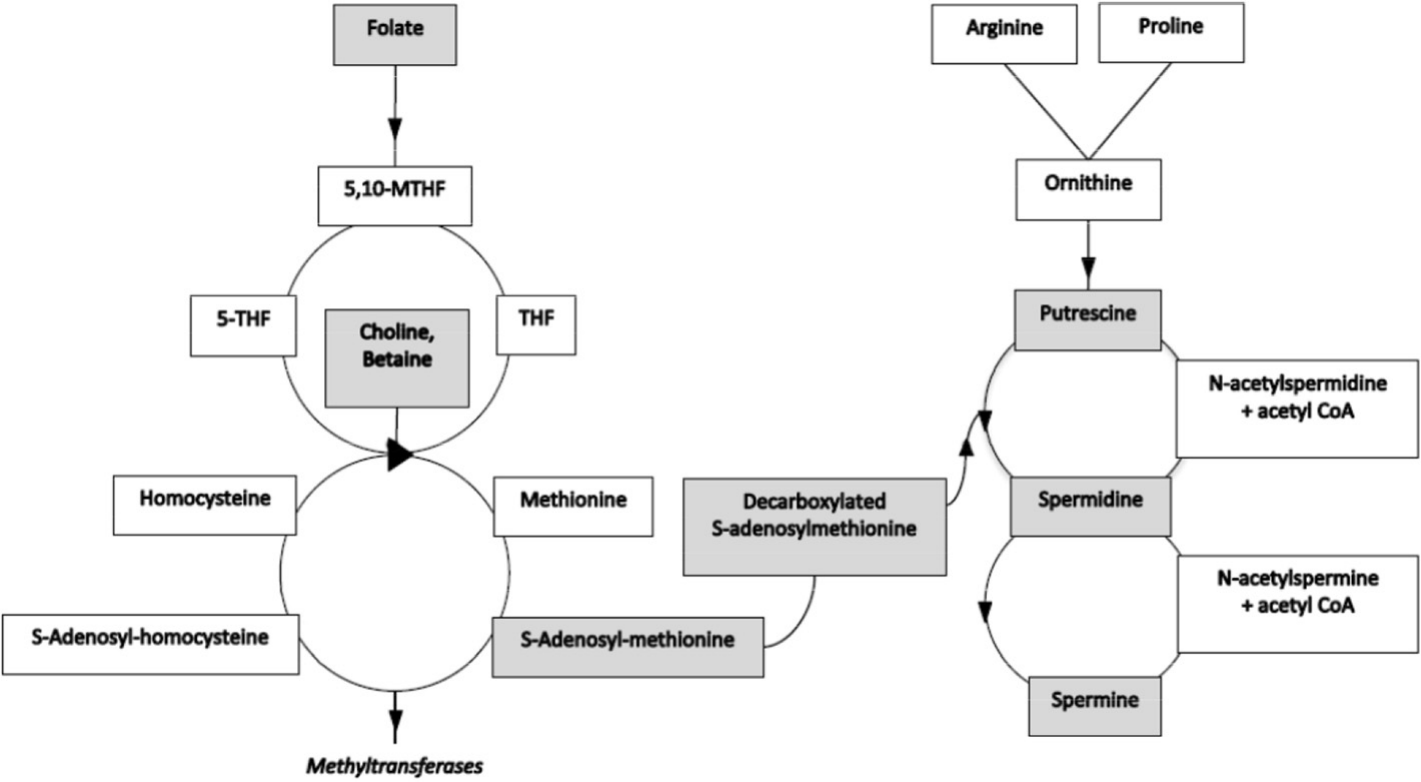
Folate, homocysteine and polyamine pathways. Gray cells highlight molecules of special interest.

FA affects many aspects of reproduction and fertility as well as imprinting and related parent-of-origin effects. Anomalies in FA metabolism can affect fertility, placental function and pregnancy in humans [12]–[17] and in mice [35]. FA acid metabolism is actively involved in DNA methylation, a major class of epigenetic modification (see Figure 3 for a schematic of the relevant pathways). The one-carbon (folate) pathway involves acquisition of a methyl group from diet or metabolic salvage, and then its transfer to S-adenosylmethionine (SAM) in the methylation (homocysteine) pathway. SAM is the one-carbon donor for methylation of nucleic acids, proteins, lipids and other molecules [9]–[11]. Methylation changes are the molecular basis for many imprinting [36] and some parent-of-origin effects [37]. FA deficiency affects expression of many genes in mouse sperm [16]. Some FA-induced epigenetic changes can also be transmitted through the germline to affect phenotypic variation in subsequent generations [38]–[41]. Recently, the egg receptor (Juno) for the sperm cell-surface protein (Izumo1) was identified [42], see also [43]. These two proteins mediate egg-sperm recognition and activate a block to polyspermy. Interestingly, Juno is a member of a folate receptor family, but does not bind folate. Whether FA affects interactions between Juno and Izumo1 has not been tested. Anomalies in FA metabolism could therefore bias allelic inheritance through imprinting and related parent-of-origin effects, but direct evidence for effects on gametes and fertilization is lacking.

An alternative hypothesis involves polyamine metabolism. This pathway plays a central role in cell proliferation, cellular reprogramming, autophagy, transcription and translation, apoptosis and necrosis not only in somatic cells but also in haploid gametes [44],[45]. Polyamines such as spermine, spermidine, putrescine and cadaverine are short chain organic molecules that possess several amines. Polyamines are highly charged molecules, with more than 90% of intracellular molecules bound to DNA and RNA. Their biosynthesis is one of the most highly regulated pathways in part because excess or deficiency can disrupt essential biological functions and because several end-products can be toxic [44],[45]. SAM is both the methyl donor for all methylation reactions as well as the substrate for spermine and spermidine biosynthesis (Figure 3). When FA and SAM are limiting, cells preserve polyamine synthesis at the expense of methylation [46],[47]. Acetyl-CoA is a co-factor in polyamine degradation. Acetyl-CoA is also used to produce choline and betaine, which serve as an alternative methyl donor (Figure 3). Thus anomalies in polyamine metabolism could affect methylation by limiting access to alternative methyl donors and by preferentially using SAM for polyamine biosynthesis rather than for methylation.

Polyamines play a prominent role in fertility and gamete function. Anomalies in polyamine levels are associated with infertility [47],[48] and dietary supplementation with SAM at least partially restores fertility [49]. Mice with transgenic over-expression of ornithine decarboxylase (ODC) are infertile [50],[51]. ODC catalyzes the first reaction in synthesis of putrescine from arginine and proline; putrescine in turn is converted to spermidine and then spermine. Polyamine activity in spermatids and spermatozoa is tightly regulated [44]. OAZ3 (ornithine decarboxylase antizyme 3 – an ODC inhibitor) is a testis-specific inhibitor of ODC1 – the rate limiting step in polyamine synthesis. OAZ3 deficient mice produce aberrant sperm that are incapable of fertilization because of defects in sperm motility [52]; OAZ3 is a potent inhibitor of ODC in spermiogenesis [53],[54]. Moreover, AZIN2, which blocks the inhibitory effects of OAZ3 on ODC, is abundant in haploid cells [55],[56]. Gene expression profiles of *Lrp6-*deficient versus wild-type mice on control versus FA-supplemented diets show differences for several genes involved in polyamine synthesis, namely *Odc1, Sat1 –* spermine/spermidine N-acetyl N1-transferase 1, and *Oaz1 -* ornithine decarboxylase antizyme 1 – another ODC inhibitor [24]. A recent study identified *Oaz1* as a differentially expressed mRNA in sperm from folate deficient mice [16]. Thus folate supplementation in certain NTD mutant mice could compromise gamete function through either methylation metabolism, polyamine biology, or both.

In summary, FA exposure led to a strong departure from Mendelian segregation, with greatly reduced numbers of mutant heterozygotes and homozygotes without changes in embryonic viability. We propose that FA supplementation in these NTD models disrupted the folate, methylation and polyamine pathways, leading to preferential fertilization and biased segregation. Surveys are needed to test for similar results among dietary responses to FA supplementation with other NTD models to determine whether similar functions and pathways are involved. Effects of FA and polyamine supplementation on gamete function and fertilization should be tested in vivo and in vitro. Finally, studies are needed to test hypotheses about the developmental and biochemical mechanisms by which dietary supplements affect NTDs, embryonic viability, gamete biology, and fertilization.

## Conclusions

In both humans and mouse models, dietary folate supplementation reduces the incidence and severity of neural tube defects, presumably by correcting developmental defects in the neural tube during embryogenesis. Tests for folate responsiveness in two NTD mouse models (in *Apob*^*tm1Unc*^ and *Vangl2*^*Lp*^) did not show a change in incidence or severity of NTDs, suggesting that these mutant mice are examples of NTD resistance to folate supplementation. Unexpectedly however we noted a biased Mendelian genotype distribution that strongly favored wild-type heterozygotes and homozygotes over mutant homozygotes. A review of the literature revealed other examples with similar biases, but these were interpreted as evidence for folate-induced embryonic lethality. Reanalysis of our results and published evidence revealed no evidence for reduced litter sizes or increased fetal resorptions in these cases. We propose that folic acid supplementation biases fertilization in favor of wild-type gametes, perhaps through folate-induced disturbances in polyamine metabolism.

## Methods

### Mice

*Apob* (B6.129P2-*Apobtm1Unc*/J; JR002053) and *Lp* (LPT/Le; JR000220) mutants were purchased from the Jackson Laboratory. All mice were raised on the PMI Nutrition Laboratory Autoclavable Rodent Diet #5010 and maintained with trio matings. Test and control studies were contemporaneous.

### Study design

**Figure 4:**
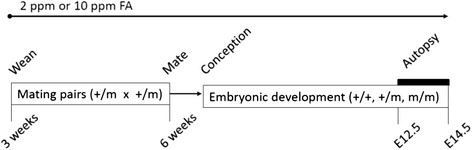
**Dietary supplementation protocol.** Three-week old female and male heterozygous mice ( or *Lp* mutants) were weaned on either the 2 ppm or 10 ppm FA diet, mated at 6 weeks of age, and then maintained on these diets through the remainder of the study. Embryos examined at E12.5 – E14.5.

Heterozygous males and females from both mutants were weaned at 3 weeks of age and thereafter maintained on either a control diet containing 2 ppm FA (D05072702, Research Diets) or a supplemented diet containing 10 ppm FA (D05072701, Research Diets) for at least 3 weeks prior to mating (Figure 4). Timed pregnancies were then generated by mating 6–10 week old females with males overnight. Upon discovery of a plug, females were kept on the same diet until they were sacrificed. Between E12.5-14.5 pregnant females were sacrificed and embryos examined (Figure 4). Tissues were obtained from all embryos for DNA extraction and genotyping. All mice shared the same animal room with controlled temperature, humidity, and 12 hour light–dark cycle. Mice were provided food and water *ad libitum*. The CWRU Institutional Animal Care and Use Committee approved all procedures.

### Special diets

The only difference between the two diets used for the supplementation study was the amount of FA, which was 5 times higher in the supplemented diet (10 ppm; D05072701, Research Diets) than the control diet (2 ppm; D05072702, Research Diets). We used 2 ppm FA because FA is required for proper breeding and fetal development based on many factors [57]. The estimated minimal FA requirement in mice is 0.5 ppm. However this concentration does not include a margin of safety [57]. Any concentration added to the diet should be higher than this minimum to account for nutrient losses during preparation and storage of the diet. In addition, a study similar to ours showed that 0 ppm FA caused embryonic lethality of *crooked-tail* mutant embryos, but a shift to the expected exencephalic phenotype at 4 ppm [33]. The percentage of affected *Cd/Cd* embryos decreased with higher concentrations of FA (7 ppm or 10 ppm), indicating that 4 ppm could serve as the control diet, thereby enabling the expected penetrance of NTDs, which was not possible with 0 ppm. At least two other studies have used this 2 ppm versus 10 ppm diet protocol [24],[25].

### Phenotype assessment

Between E12.5-14.5, pregnant females were sacrificed and the embryos examined for NTDs, looped tail and resorptions. Resorptions were counted as dead embryos, including those that appeared only as ‘dark spots’ (necrosis) in the uterus. Resorptions were not genotyped. Litter size was counted as the number of live embryos at autopsy. The number of corpora lutea was not counted.

### Genotyping

Genotyping for *Apob* was done according to the protocol provided by the Jackson Laboratory. The *Lp* genotyping protocol was previously described [58].

### Statistical analysis

Statistical comparisons using the chi-square and Fisher’s exact tests, as appropriate, were performed using GraphPad QuickCalcs Web site: http://graphpad.com/quickcalcs/chisquared1.cfm and http://graphpad.com/quickcalcs/contingency1.cfm.

Fisher’s exact test was used to evaluate differences in the incidence of NTDs in mutant homozygotes between the 2 ppm versus 10 ppm diets (Table 1). Chi-square goodness-of-fit tests (2 df) were used to test for departures from Mendelian expectations for the two NTD models on the 2 ppm or 10 ppm diets (Tables 2 and 3). We used both the statistical P value as well as the magnitude of the phenotype effect to assess results.

## Competing interests

The authors declare no competing interests.

## Authors’ contributions

GAN performed the work, GAN and JHN conceived the study, planned the work, analyzed the data, and wrote the paper. Both authors read and approved the final manuscript.
